# In Vitro Evaluation of the Photoreactivity and Phototoxicity of Natural Polyphenol Antioxidants

**DOI:** 10.3390/molecules27010189

**Published:** 2021-12-29

**Authors:** Brandon Aguiar, Helena Carmo, Jorge Garrido, José M. Sousa Lobo, Isabel F. Almeida

**Affiliations:** 1 UCIBIO/REQUIMTE, Medtech Laboratory of Pharmaceutical Technology, Department of Drug Sciences, Faculty of Pharmacy, University of Porto, 4050-313 Porto, Portugal; brandonlageaguiar@gmail.com (B.A.); slobo@ff.up.pt (J.M.S.L.); 2 UCIBIO/REQUIMTE, Laboratory of Toxicology, Department of Biological Sciences, Faculty of Pharmacy, University of Porto, 4050-313 Porto, Portugal; helenacarmo@ff.up.pt; 3 CIQUP, Department of Chemical Engineering, School of Engineering (ISEP), Polytechnic of Porto, 4200-072 Porto, Portugal

**Keywords:** photosafety, photoreactivity, phototoxicity, polyphenols, natural phenolic antioxidants, reactive oxygen species, keratinocytes, skin care, cosmetic products

## Abstract

Polyphenols are a large family of natural compounds widely used in cosmetic products due to their antioxidant and anti-inflammatory beneficial properties and their ability to prevent UV radiation-induced oxidative stress. Since these compounds present chromophores and are applied directly to the skin, they can react with sunlight and exert phototoxic effects. The available scientific information on the phototoxic potential of these natural compounds is scarce, and thus the aim of this study was to evaluate the photoreactivity and phototoxicity of five phenolic antioxidants with documented use in cosmetic products. A standard ROS assay was validated and applied to screen the photoreactivity of the natural phenolic antioxidants caffeic acid, ferulic acid, *p*-coumaric acid, 3,4-dihydroxyphenylacetic acid (DOPAC), and rutin. The phototoxicity potential was determined by using a human keratinocyte cell line (HaCaT), based on the 3T3 Neutral Red Uptake phototoxicity test. Although all studied phenolic antioxidants absorbed UV/Vis radiation in the range of 290 to 700 nm, only DOPAC was able to generate singlet oxygen. The generation of reactive oxygen species is an early-stage chemical reaction as part of the phototoxicity mechanism. Yet, none of the studied compounds decreased the viability of keratinocytes after irradiation, leading to the conclusion that they do not have phototoxic potential. The data obtained with this work suggests that these compounds are safe when incorporated in cosmetic products.

## 1. Introduction

Polyphenols (PPs) constitute one of the most numerous and widely distributed groups of natural products in the plant kingdom. The chemical structure of PPs is characterized by the presence of one or more phenolic hydroxyl groups, bound to one or more benzene ring systems [1]. Polyphenols exhibit a variety of beneficial biological activities in humans, including antiviral, antibacterial, anticarcinogenic, hepatoprotective, anti-inflammatory, and antioxidant action [2,3,4,5]. As antioxidants, polyphenols may protect cell constituents against oxidative damaging effects of reactive oxygen species (ROS), such as singlet oxygen, superoxide, and hydroxyl radicals, limiting the risk of several diseases associated with oxidative stress [6,7]. Due to their chemical properties, PPs are able to scavenge ROS and chelate transition metals ions, such as iron and copper, which has attracted the interest of the cosmetic industry for their use in skin care formulations [8,9,10]. Ultraviolet (UV) exposure is one of the main factors inducing skin cancer, and cutaneous cells may be damaged directly by UV radiation or indirectly by UV-mediated ROS overproduction [11]. Experimental and epidemiologic studies have suggested that polyphenols protect the skin from the adverse effects of UV radiation through multiple pathways [12]. Therefore, several compounds belonging to this family are already used as ingredients in a number of commercial cosmetic products available on the market [13].

Increased consumer demands for natural cosmetics urged the industry to develop formulations using natural extracts with active ingredients, such as polyphenols, as a promising and effective solution for skin care [13]. However, the chromophores present in polyphenols’ structure have the ability to absorb UV/Vis radiation and undergo chemical reactions resulting in a cascade of events that may result in phototoxic reactions [14]. Phototoxicity is defined as a toxic response elicited by topically or systemically administered photoreactive chemicals after the exposure of the body to environmental light [15]. Active pharmaceutical ingredients and excipients for systemic administration, clinical formulations for topical application, dermal patches, and others, have phototoxic potential and can cause notable phototoxic reactions. Consequently, regulatory agencies, US FDA, EU EMA, and ICH, provide photosafety guidelines, introducing test methods and evaluation strategies [15,16,17].

Safety assessment of cosmetic products is mandatory according to the European Union legislation [18]. The required safety evaluation includes relevant toxicological studies on cosmetic ingredients, including a photo-induced toxicity evaluation [18]. Since animal testing has been banned for cosmetic products, a number of in vitro photosafety tests have been proposed over the past years, including UV spectrum analysis, a 3T3 Neutral Red Uptake phototoxicity test (3T3 NRU PT), and a reactive oxygen species (ROS) assay [18,19,20].

The ROS assay was designed for photoreactivity assessment of drugs, the principle of which is monitoring photochemical reactions in the test chemicals exposed to simulated sunlight [19,20]. Through these photochemical reactions ROS, such as superoxide anion and singlet oxygen, can be generated, and these photochemical processes can be a trigger for drug-induced phototoxicity [19,20]. High levels of ROS can cause cytotoxicity through damage of DNA, lipids, and proteins by oxidative stress.

The in vitro methodology 3T3 NRU-PT uses the mouse fibroblasts cell line Balb/c 3T3 and neutral red uptake as the endpoint of cytotoxicity. The phototoxicity is then determined by the relative reduction in the viability of the cells exposed to the test chemical in the presence and the absence of light simulating the sunlight. Studies reported in literature concluded that this test is over-sensitive, mistakenly predicting animal and human photo safety risks, leading to a high number of false positives when compared to in vivo results [21,22,23]. Considering this limitation, a modification of the 3T3 NRU-PT methodology was proposed by our group based on the use of a human keratinocyte cell line (HaCaT) [24]. Since this methodology uses human keratinocyte cells, it represents a more realistic model, given that these are the most abundant type of cells present in the external layer of the skin, where topical compounds are applied and exposed to sunlight radiation [24].

**Figure 1:**
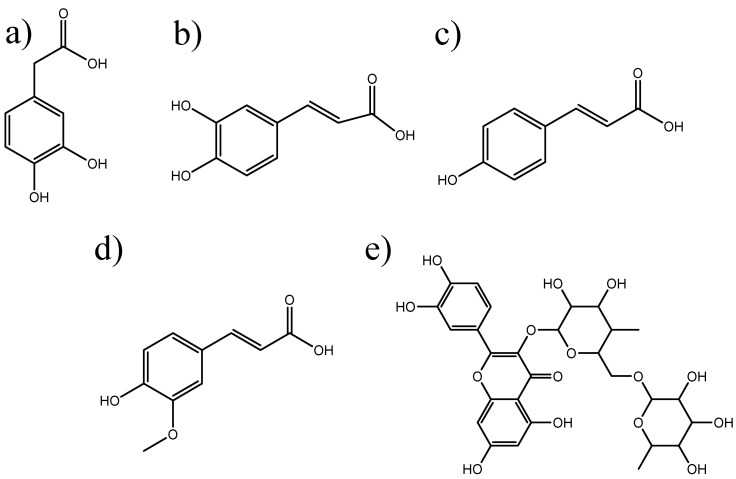
Chemical structures of the natural polyphenols tested. (**a**) 3,4-Dihydroxyphenylacetic acid (DOPAC); (**b**) caffeic acid; (**c**) *p*-coumaric acid; (**d**) ferulic acid; (**e**) rutin.

The aim of the present study was to estimate the photo-induced toxicity potential of the natural polyphenols *p*-coumaric acid, caffeic acid, 3,4-dihydroxyphenylacetic acid (DOPAC), ferulic acid, and rutin (Figure 1), which are already used as cosmetic ingredients or are being considered due to their interesting chemical and antioxidant properties [25,26,27]. A ROS assay was validated and implemented for the exploratory photosafety assessment of the compounds, and their cytotoxicity and phototoxicity were further assessed using a human keratinocyte cell line (HaCaT).

## 2. Results and Discussion

The initial consideration for the assessment of photoreactive potential is whether a compound absorbs photons at any wavelength between 290 and 700 nm. A compound that has a molar extinction coefficient (MEC) greater than 1000 L mol^−1^ cm^−1^ at any wavelength between 290 and 700 nm is considered to be sufficiently photoreactive to result in direct phototoxicity [15].

**Figure 2:**
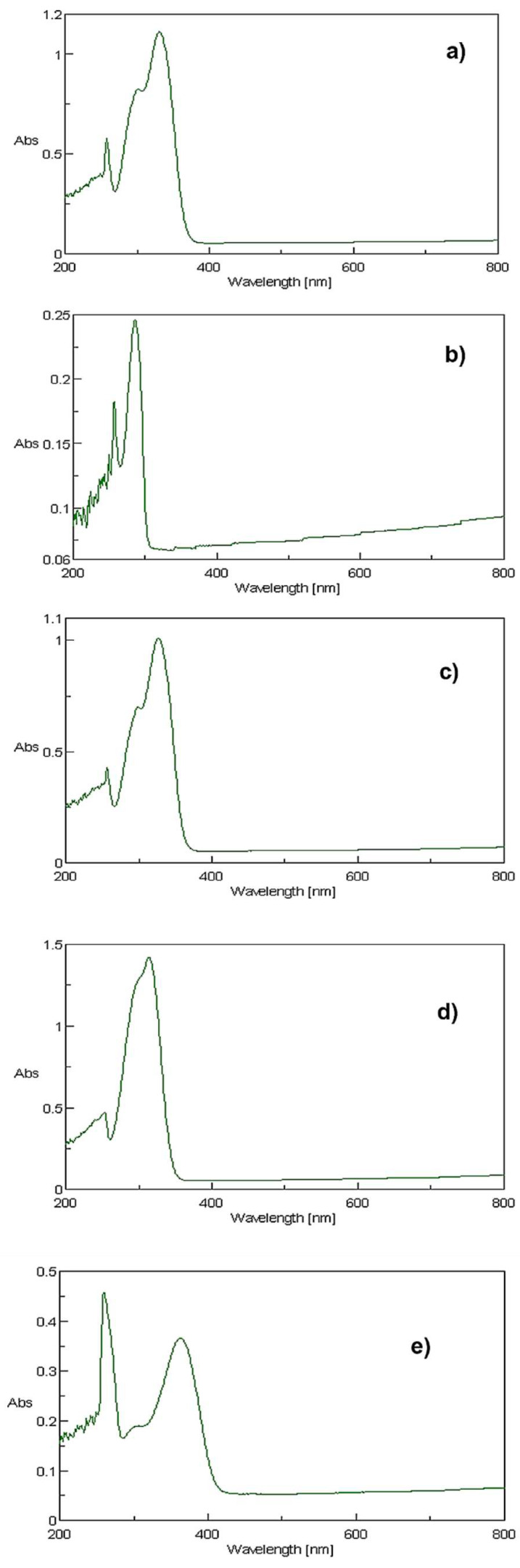
Absorption spectra obtained for each compound dissolved in DMSO at a concentration of 10 µg/mL. (**a**) Caffeic acid; (**b**) DOPAC; (**c**) ferulic acid; (**d**) *p*-coumaric acid, and (**e**) rutin.

The absorption spectra of caffeic, *p*-coumaric, and ferulic acids, DOPAC, and rutin in DMSO at UV-visible light are shown in Figure 2. 

All the compounds absorb in the spectral range of 200 to 700 nm, with maximum wavelength at or above 290 nm and with MEC typically greater than 4000 L mol^−1^ cm^−1^ (Table 1). Polyphenols are biological compounds containing π conjugated systems with phenolic hydroxyl groups. The π type molecular orbitals’ electronic transitions are responsible for the UV-visible spectrum of this group of compounds [28].

All the compounds tested presented MEC higher than 1000 L mol^−1^ cm^−1^, which means that all are possible phototoxic compounds worth studying [15].

Prior to performing the ROS assay, the solar simulator was evaluated, and the experimental conditions were optimized to ensure that measured values of singlet oxygen (SO) and superoxide anion (SA) were close to those mentioned in the literature [29]. The optimization of the ROS generation assay was performed using positive and negative controls and a feasibility study was conducted using reference chemicals. Under the experimental conditions used, all substances tested in the feasibility study met the acceptance criteria giving values for SO and SA within the admissible value ranges [29].

The ROS assay was carried out for the polyphenolic compounds, and the capacity of the tested substances to generate ROS, at the concentration of 200 μM, is shown in Table 2.

The results obtained show that, with the exception of DOPAC, all compounds, can be classified as non-photoreactive. Although these substances showed a UV-visible light absorption and MEC higher than 1000 L mol^−1^ cm^−1^ they did not generate ROS under the tested conditions, either SO or SA species. Surprisingly, DOPAC was able to induce the generation of SO species and, therefore, was classified as photoreactive, despite this compound having the lowest MEC value in the UV-visible range among all the compounds studied. More studies are needed to understand the results obtained for DOPAC, which may also be important, in the near future, to establish a correlation between the chemical structure of a compound and its ability to be photoreactive.

The cytotoxicity of DMSO used for the evaluation of phototoxic effects, in the presence and absence of irradiation, after 1h of exposure was evaluated. For the non-irradiated plate, DMSO did not show a statistically significant difference relative to the negative controls. However, for the irradiated plate, there was a significant difference between the 1% DMSO and the solvent control relative to the negative controls (*p* < 0.0001), which justified the use of solvent control on all experiments to guarantee that the differences in cell viability were only attributed to the compounds under study. To ensure the feasibility of the phototoxicity assay using a human keratinocyte cell line (HaCaT), 5-methoxypsoralen, chlorpromazine hydrochloride, and quinine were tested as positive controls, and acetylsalicylic acid, hexachlorophene, and sodium lauryl sulfate as negative controls [24]. 

**Figure 3:**
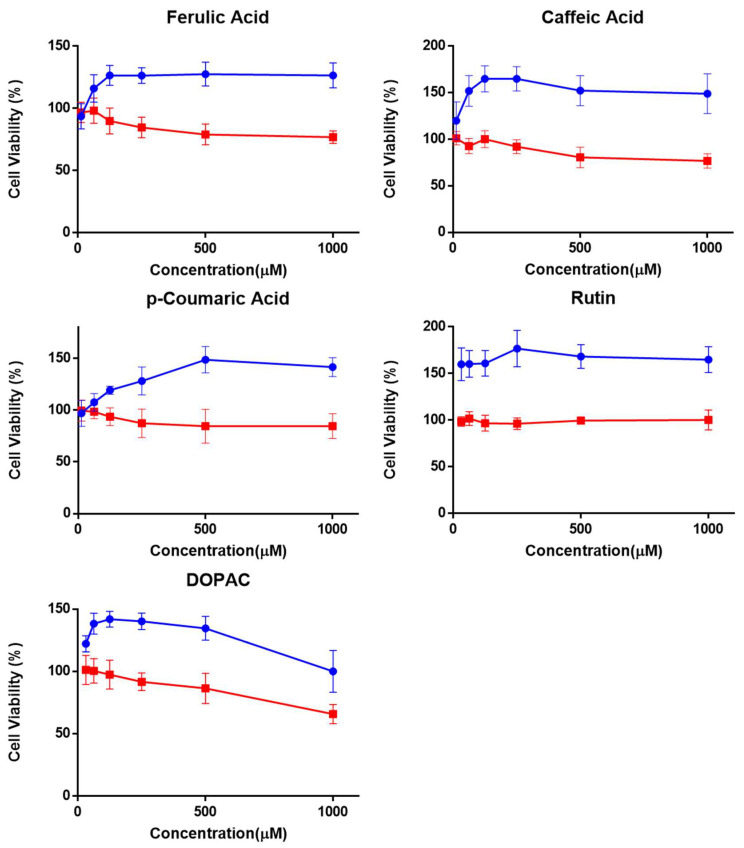
Cell viability of HaCaT cells exposed to the tested compounds assessed using the Neutral Red assay. (▬) Irr−: Non-irradiated; (▬) Irr+: Irradiated plate. Data are presented as mean ± SD (*n* = 3) relative to solvent control.

The results obtained from the phototoxicity assay comparing the cell viability of the HaCaT cells, irradiated and non-irradiated, in the presence of the tested (poly)phenolic compounds are depicted in Figure 3.

The ranges of concentrations of the chemicals tested in the presence (Irr+) and the absence (Irr−) of light were determined in dose range-finding experiments, considering the maximum concentration of 1000 µM. A geometric dilution series was used and adjusted, when necessary, as a function of concentration–response in the presence and absence of irradiation. Within the tested concentration range (12.5; 31.25; 62.5; 125; 250; 500, and 1000 µM) none of the test substances induced a 50% decrease in cell viability, therefore, it was not possible to calculate the corresponding IC_50_ and PIF values. On the contrary, it was possible to perceive a dose-dependent increase in viability for the irradiated cells in the presence of the tested substances, possibly due to the photoprotective effects of these antioxidants against the oxidative damage induced by the radiation leading to a higher percentage of viable cells when compared to the control (untreated irradiated cells). It is described in the literature that UV radiation leads to the generation of ROS, an overproduction of nitric oxide, and depletion of antioxidant defenses in keratinocytes [30]. For these reasons, polyphenols with antioxidant ability have been studied as photoprotective agents. Although most studies focused on the photoprotective capacity of polyphenols, they did not study their phototoxic potential. Previous studies confirmed the ability of caffeic, ferulic, and *p*-coumaric acids to scavenge ROS and reactive nitrogen species (RNS). Moreover, protection against the deleterious effects of UV radiation was also established in vivo or skin cells for these three phenolic compounds [31,32,33,34]. These data may explain the increase in the percentage of viable cells when exposed to irradiation in the presence of the compounds under study. Rutin, like caffeic, ferulic, and *p*-coumaric acids tested negative in the ROS generation assay and did not induce phototoxicity in the HaCaT cell line. There is some contradictory information in the literature regarding rutin’s phototoxic potential, hence the interest of the results obtained. The evaluation of the phototoxic potential using a keratinocyte cell system (HaCaT), showed that rutin demonstrated phototoxicity [35]. On the contrary, using an experimental setup that employs capillary electrophoresis with electrochemical and UV detection to test phototoxicity of plant extracts and components in terms of oxygen consumption and generation of reactive oxygen species upon irradiation with visible light, it was possible to conclude that rutin was not phototoxic [36], which was in agreement with the results obtained in this work where rutin did not show photoreactivity as it did not generate either SO nor SA in the ROS generation assay. On the other hand, in this work, it was also shown that rutin by itself presents no phototoxic potential in the HaCaT cell line. These contradictory findings highlight the importance of using standardized testing conditions and also the use of an appropriate light source in order to avoid misleading results. Interestingly, and despite the structural similarity between DOPAC and the other PPs studied, DOPAC generated SO in the ROS generation assay, and was classified as photoreactive. However, when tested in the HaCaT cell line, DOPAC showed to be non-phototoxic. From the results obtained, DOPAC appears to be photoreactive but not phototoxic, so it is not expected that phototoxic reactions occur after the topical application of this compound.

## 3. Materials and Methods

### 3.1. Reagents

3,4-Dihydroxyphenylacetic acid (DOPAC), caffeic acid, trans-ferulic acid, *p*-coumaric acid, rutin, chlorpromazine hydrochloride, di-sodium hydrogen phosphate dodecahydrate, sodium phosphate monobasic monohydrate, Neutral Red (NR), and dimethyl sulfoxide (DMSO) were purchased from Sigma–Aldrich (Madrid, Spain). Quinine hydrochloride, benzocaine, diclofenac, and erythromycin were purchased from Acofarma (Madrid, Spain). The immortalized human keratinocyte (HaCaT) cell line was obtained from Cell Lines Service (CLS) (Eppelheim, Germany). Dulbecco’s Modified Eagle Medium (DMEM) with 4.5 g/L d-glucose, l-glutamine, 25 mM HEPES, and DMEM with 4.5 g/L d-glucose, l-glutamine, 25 mM HEPES with no phenol red, Dulbecco’s Phosphate Buffered Saline (DPBS), Fetal Bovine Serum (FBS), and trypsin EDTA were purchased from Gibco Life Technologies (Waltham, MA, USA). Ethanol was supplied by Aga (Lisbon, Portugal). *N*,*N*-Dimethyl-4-nitrosoaniline (RNO), imidazole, and Nitro Blue Tetrazolium (NBT) were purchased from Alfa Aesar (Kandel, Germany). 

### 3.2. Spectral Absorption

The absorption spectrum of each studied compound was determined in the range of 290 to 700 nm, according to OECD Test Guideline 101, using a Jasco V650 UV/VIS spectrophotometer [37]. The substances were dissolved in DMSO in order to obtain a final concentration of 10 μg/mL and the absorption spectra were measured using UV-transparent quartz cuvettes (path length = 10 mm). Each spectrum was corrected for solvent-specific baseline absorption. Molar extinction coefficients (MEC) were calculated using the highest absorption peaks from 290 to 700 nm [15]. 

### 3.3. Reactive Oxygen Species (ROS) Assay

The ROS assay protocol was established, and the validation studies were conducted according to the procedure described in the literature [19,29]. Stock solutions of all tested substances were prepared at 10 mM concentration in DMSO and used within the same day, protected from light. Briefly, singlet oxygen (SO) generation was detected by spectrophotometric measurement of *p*-nitrosodimethylaniline (RNO) bleaching at 440 nm using imidazole as a selective acceptor of singlet oxygen. Samples, containing the tested chemical (200 μM), RNO (50 μM), and imidazole (50 μM) in 20 mM sodium phosphate buffer (NaPB, pH 7.4), were placed in a tube and mixed with a vortex mixer and sonicated, protected from light, for 10 min. The mixture was transferred into a Hellma quartz glass high-performance cell and checked for precipitation under a microscope before light exposure. Then, the samples were irradiated using a Fitoclima S600PL thermostatic solar simulator (Aralab, Portugal), equipped with eight Repti Glo (20 W) UV-Vis lamps, for 90 min at 25 °C. After irradiation, the absorbance was read again at 440 nm. Superoxide anion (SA) generation was detected by observing the reduction of nitroblue tetrazolium (NBT) to monoformazan (NBT+), the formation of which can be monitored spectrophotometrically at 560 nm. Samples containing the tested compounds (200 μM) and NBT (50 μM) in 20 mM NaPB were irradiated, and the reduction in NBT was measured by the increase in absorbance at 560 nm in the same manner as for SO determination. Experiments were performed in triplicate.

Since the solar simulator used was different from the recommended models, it was necessary to validate the irradiation conditions. A ROS assay was performed to ensure irradiation conditions satisfied the recommended criteria using positive (quinine) and negative controls (sulisobenzone) and reference chemical compounds [29].

According to the result (mean of triplicate determinations) from the ROS assay, the tested polyphenolic compounds were classified as photoreactive substances when an SO value 25 or more and/or an SA value of 20 or more was measured; in turn, it was determined as a non-photoreactive substance when values of less than 25 for SO and less than 20 for SA were recorded [29].

### 3.4. Cell Culture

The HaCaT cells were maintained at 37 °C in a humidified atmosphere of 95% air and 5% CO_2_ in the incubator in DMEM with 10% FBS and 1% antibiotics. Using an inverted microscope, cell confluence was observed and if the cells reached 70–80% confluence, subculture was done to prevent cell death. For this purpose, the culture medium was aspirated, and the cells were washed with DPBS, 2 mL of trypsin 0.25% was added and incubated for 7 to 8 min at 37 °C in a 5% CO_2_ atmosphere. After cells detached, a fresh medium was added to block the trypsin action. For cell counting, a 10 µL of cell suspension was placed in a Neubauer chamber where the cells were counted. The obtained cell suspension was then subdivided into new flasks with a fresh cell culture medium. For cell freezing, DMSO (5% *v*/*v*) was used as a cryo-preservative to prevent the formation of crystals during the storage phase. In order to determine the time necessary for the cells to duplicate, 1 × 10^6^ cells were seeded in five 75 cm^2^ flasks and incubated for 24 h at 37 °C in a 5% CO_2_ atmosphere to reach complete adherence. Then, the cells of each flask were counted at different times. The results were plotted in a graphic representing cell number versus time, from which the doubling time was calculated using linear regression analysis. The calculated doubling time obtained was 20.43 h, which is consistent with the values reported in the literature, thus confirming that the cells used were in normal growth conditions [38].

### 3.5. Phototoxicity Assay

For the study of phototoxicity, a previous protocol implemented in our laboratory was followed [24].

The height of the UVA/UVB Osram lamp (240V E27) was adjusted in order to irradiate the cells with an irradiation UVA dose of 1.7 mW/cm^2^ (Cosmedico radiometer, UVM-7), according to OECD guideline [23]. During irradiation (10 min), the plates were kept inside a styrofoam recipient containing a water-cooling system, and the temperature was monitored throughout the procedure. 

Briefly, to perform the NR uptake assay, HaCaT cells were seeded (2 × 10^4^ cells/well) and incubated at 37 °C in a 5% CO_2_ atmosphere for 24 h. Afterward, the medium was removed, and different concentrations of the test substances were added, and cells were incubated under the same conditions for 1 h. One plate was kept in the dark while the other plate was irradiated for 10 min with the temperature kept at 29–32 °C. Afterward, the cells medium was replaced with fresh DMEM without phenol red and incubated for 18–22 h. After this incubation period, cells from both plates were washed with DPBS and complete DMEM containing 50 μg/mL NR was added to each well and incubated for 3 h. After incubation with NR, the NR solution was removed, and a NR desorb solution (50% ethanol:1% acetic acid:49% distilled water) was added to extract the NR dye from the cells. For the reading procedure, the absorbance was measured at 540 nm. Within each plate, DMSO controls were tested. Cell viability data obtained from each plate was expressed as the absorbance ratio of treated cells to solvent control cells and was further used to estimate the IC_50_ values using linear regression analysis. The Photo-Irritation-Factor (PIF) value for each test substance was calculated as the ratio between the IC_50_ value of the irradiated (Irr+) versus the IC_50_ value of the non-irradiated (Irr−) cells. According to the OECD guideline, a PIF index lower than 2 predicts the absence of a phototoxic effect, a PIF index between 2 and 5 predicts a probable phototoxic effect, and a PIF higher than 5 predicts a phototoxic effect [23].

Tested compounds were evaluated in a range of concentrations: 12.5; 31.25; 62.5; 125; 250; 500, and 1000 µM.

### 3.6. Statistical Analysis

All data are presented as mean ± standard deviation (SD) from at least three independent experiments run in triplicate. To confirm the normality and homogeneity of variance, D’Agostino–Pearson omnibus normality test was used and afterward the One-way analysis of variance (ANOVA), followed by Dunnett’s post hoc test (comparison to negative control cells with solvent), was performed. The graphs were generated with the software GraphPad Prism for Windows (version 6.0, GraphPad Software, Inc. (San Diego, CA, USA).

## 4. Conclusions

In this study, a Reactive Oxygen Species (ROS) assay and a 3T3 Neutral Red Uptake Phototoxicity assay (3T3 NRU-PT) were used to examine the behavior of natural phenolic antioxidants when exposed to radiation mimicking the sunlight in order to examine their photoreactivity and phototoxicity potential. 

The results obtained allowed the conclusion that, although rutin and caffeic, *p*-coumaric, and ferulic acids absorb UV-visible light radiation and present MEC higher than 1000 L mol^−1^ cm^−1^, they are classified as non-photoreactive. Moreover, these compounds did not induce phototoxicity when tested using the HaCaT cell line. The data found suggest that these antioxidants by themselves are not expected to cause phototoxicity and thus they can be considered safe for use in cosmetic formulations. On the other hand, it was possible to observe an increase in the cell’s viability exposed to the radiation when in the presence of these antioxidants revealing a possible photoprotective effect that could be interesting to study to support their use as possible photoprotective agents in cosmetic formulations. 

In the case of DOPAC, this compound showed to be photoreactive, although no phototoxicity was observed in the 3T3 Neutral Red Uptake assay. However, more studies should be conducted to understand the mechanisms behind DOPAC photoreactivity, to ensure its photosafety, and also to comprehend the role and relationship between the chemical structure and the potential of a compound to be photoreactive.

## Figures and Tables

**Table 1:** Molar extinction coefficient (MEC) and maximum wavelength of the tested compounds at 10 µg/mL.

Compounds	λmax (nm)	Absorbance	MEC (L mol^−1^ cm^−1^)
DOPAC	286	A_286_ = 0.246	ℇ_286_ = 4129
Caffeic acid	331	A_331_ = 1.112	ℇ_331_ = 20,036
*p*-Coumaric acid	314	A_314_ = 1.421	ℇ_314_ = 23,335
Rutin	363	A_363_ = 0.366	ℇ_363_ = 22,354
Ferulic acid	327	A_327_ = 1.011	ℇ_327_ = 19,635

**Table 2:** Results obtained for the tested compounds using the ROS assay.

Compounds	^1^ $O_{2}$	$O_{2}^{•-}$	Photoreactivity
Caffeic Acid	−2	4	Non-photoreactive
Ferulic Acid	−9	−1	Non-photoreactive
*p*-Coumaric Acid	−3	2	Non-photoreactive
DOPAC	38	4	Photoreactive
Rutin	15	12	Non-photoreactive

## Data Availability

All the data presented in this study is contained within the article.
